# Digital Health Literacy of the Population in Germany and Its Association With Physical Health, Mental Health, Life Satisfaction, and Health Behaviors: Nationally Representative Survey Study

**DOI:** 10.2196/48685

**Published:** 2024-02-21

**Authors:** Lars König, Adelheid Kuhlmey, Ralf Suhr

**Affiliations:** 1 Institut für Medizinische Soziologie und Rehabilitationswissenschaft Charité – Universitätsmedizin Berlin Berlin Germany; 2 Stiftung Gesundheitswissen Berlin Germany

**Keywords:** digital health, digital health literacy, eHealth, eHealth literacy, health behaviors, health literacy, life satisfaction, mental health, physical health, representative survey

## Abstract

**Background:**

Digital health literacy, also known as eHealth literacy, describes the ability to seek, find, understand, and apply health information from the internet to address health problems. The World Health Organization calls for actions to improve digital health literacy. To develop target group–specific digital health literacy interventions, it is necessary to know the digital health literacy of the general population and relevant subgroups.

**Objective:**

This study aims to representatively assess the digital health literacy of the population in Germany and relevant subgroups. The results are meant to facilitate the development of target group–specific digital health literacy interventions. Additionally, this study further explores the associations between digital health literacy and physical health, mental health, life satisfaction, and diverse health behaviors.

**Methods:**

Study participants were drawn from a representative panel of the German-speaking population with internet access. To further increase the representativeness of the sample, survey weights were calculated using an iterative proportional fitting procedure. Participants answered a series of questionnaires regarding their digital health literacy, physical health, mental health, life satisfaction, and diverse health behaviors. Two-sided independent sample *t* tests were conducted to determine the significant differences between societal subgroups. Pearson correlation coefficients were calculated to explore the correlates of digital health literacy.

**Results:**

Digital health literacy is unevenly distributed within German society. The results of this study suggest that people with a low level of formal education and people with a low social status would benefit from digital health literacy interventions that address their competencies in the domains of information seeking and information appraisal. Furthermore, the results suggest that older people would likely benefit from digital health literacy interventions that address their competencies in the domains of information seeking and also information appraisal. Regarding sex, this study suggests that men might benefit from digital health literacy interventions that specifically address their competencies in the domain of information seeking. Furthermore, digital health literacy is weakly positively correlated with physical health, mental health, life satisfaction, exercise routines, fruit consumption, and vegetable consumption.

**Conclusions:**

Overall, the results of this study demonstrate that digital health literacy is associated with diverse health outcomes and behaviors. Furthermore, the results provide a starting point for the development of target group–specific digital health literacy interventions.

## Introduction

Digital health literacy, also known as eHealth literacy, can be defined as “the ability to seek, find, understand, and appraise health information from electronic sources and apply the knowledge gained to addressing or solving a health problem” [1]. Nowadays, these skills seem particularly important for two reasons. First, people from around the world regularly use the internet to acquire health information [2,3]. Second, when searching the internet for health information, people are often confronted with misinformation [4,5]. Furthermore, recent studies have found that evaluating the reliability of health information on the internet is not just difficult for the general population but also for better educated and younger subgroups like university students [6-8].

Besides its central role in the context of evaluating health information on the internet, digital health literacy seems important because it is associated with various health intentions and behaviors [9]. Studies in diverse contexts have shown, for example, that people with high digital health literacy demonstrate better physical exercise routines [10], eat more nutritionally balanced diets [11], and protect themselves better against viruses [12]. Furthermore, they have better cancer screening practices [13], are more confident about finding cancer information [14], and are less likely to fall prey to conspiracy beliefs [12]. Given these potential positive health effects, it is no surprise that the World Health Organization calls for actions to improve digital health literacy [15].

To develop target group–specific digital health literacy interventions, it is necessary to know the digital health literacy of the general population and relevant subgroups. Various instruments have been developed to measure digital health literacy [16]. One of the most widely used instruments is the eHealth Literacy Scale [17], which has already been translated into diverse languages, including Dutch [18], Chinese [19], and Korean [20]. A German version of the eHealth Literacy Scale was published in 2014 [21]. This instrument, however, was recently criticized because of content-related and methodological shortcomings, and a revised German eHealth Literacy Scale was published in 2022 [9].

This study aims to representatively assess the digital health literacy of the population in Germany and relevant subgroups, using the revised German eHealth Literacy Scale [9]. The results are meant to facilitate the development of target group–specific digital health literacy interventions. Additionally, this study further explores the associations between digital health literacy and physical health, mental health, life satisfaction, and diverse health behaviors (exercise routines, fruit consumption, vegetable consumption, soft drink consumption, alcohol consumption, cigarette consumption, and drug consumption).

## Methods

### Ethical Considerations

Before data collection, a detailed study protocol that included information about the procedures, measures, and statistical analyses was submitted to the ethics committee of the Berlin Medical Association. The ethics committee consisted of 2 medical doctors, a lawyer, a statistician, and a layperson. The ethics committee had no ethical or professional objections to the study protocol (reference Eth-39/22). Before the study started, participants provided their informed consent to take part in the study. Participants had the opportunity to opt out of the study at any time during the study. The independent, nonprofit foundation Stiftung Gesundheitswissen did not compensate the participants for their participation, and participants were informed that the foundation would only receive anonymized data.

### Survey Methodology

The market research Institute Forsa Gesellschaft für Sozialforschung und Statistische Analysen mbH (Forsa) was responsible for data acquisition [22]. The survey was conducted using the representative web-based panel forsa-omninet, which is a representative panel for the German-speaking population with internet access and currently has around 100,000 participants. A continuous recruiting process adds new participants to the panel every month. In addition, the composition of the panel is continuously monitored based on key characteristics (eg, region, age, and sex), and recruitment is adjusted accordingly. Data acquisition took place from September 22 to October 12, 2022. A random sample was drawn from the representative web-based panel. All panelists selected for the survey were invited via email. The invitation email provided information on the topic and purpose of the survey. If necessary, the selected panelists were reminded about their participation in 2 further emails. In total, 3927 panelists were invited to take part in the survey. All responses were checked for plausibility, and a comprehensive speeder analysis was conducted based on the response times. A total of 2000 panelists completed the survey. This corresponds to a response rate of about 50.9% (2000/3927), which is quite high for a relatively long survey that is completed on a voluntary basis. On average, panelists took 20 minutes to finish the survey. The sample characteristics of the participants who were invited to participate in the study (invited sample) and the participants who were included in the data analyses before (unweighted sample) and after the weighting procedure (weighted sample) can be found in the Results section. Please note that the unweighted and weighted samples are based on the 1996 study participants who answered all items from the revised German eHealth Literacy Scale. Furthermore, please note that due to sample weighting and rounding, sample sizes may vary, and percentages may exceed or fall below 100%. There are differences between these groups. For example, 50.6% (1988/3927) of the invited sample were men, but only 47.8% (955/1996) of the unweighted sample were men, which might indicate a selection bias. To correct for such response differences and to make the sample more representative, a weighting procedure was implemented. After the weighting procedure, 49% (978/1997) of the sample were counted as men. It is essential to bear in mind that a weighting procedure cannot take every existing human factor into account. Hence, there will still be differences after the weighting procedure. Nevertheless, the weighting procedure was designed to make the sample more representative. Interested readers can find detailed information about the weighting procedure in the Survey Weights section. Furthermore, the problem of nonresponders and potential response bias within the sample will be discussed in the Limitations section. It is essential to bear in mind that this study used a cross-sectional study design, and therefore no causal inference can be drawn [23]. Furthermore, participants needed basic technical skills and access to the internet to participate in the study. Therefore, this study can only be representative of the German population with internet access.

### Survey Weights

To increase the representativeness of the sample, survey weights were provided by Forsa. The survey weights were calculated using an iterative proportional fitting procedure, which allows the fitting to given structures for several characteristics with a single weighting factor for each case. The weighting of the sample was based on data from the Federal Statistical Office of Germany (Bevölkerungsfortschreibung des statistischen Bundesamts, December 31, 2020). The following aspects were used for calculating the weighting factors for each case: (1) sex in combination with 4 age groups (16-29 years, 30-45 years, 46-64 years, and 65 years and older) and region (East without Berlin or West including Berlin) and (2) federal state (Bundesland).

### Measures

#### Digital Health Literacy

The revised German eHealth Literacy Scale was used to assess digital health literacy [9]. The instrument consists of 8 items that comprise 2 subscales assessing competencies in the domain of information seeking (4 items; eg, I know how to find helpful health resources on the Internet) and information appraisal (4 items; eg, I have the skills I need to evaluate the health resources I find on the Internet). Participants rated all items on scales ranging from 1 (strongly disagree) to 5 (strongly agree). A total score was generated for each subscale by calculating the mean.

#### Physical Health and Mental Health

Physical health was assessed by asking participants “Overall, how do you currently rate your physical health?” Mental health was assessed by asking participants “Overall, how do you currently rate your mental health?” Participants answered the questions on scales ranging from 0 (very bad) to 10 (very good).

#### Life Satisfaction

The 1-item General Life Satisfaction Short Scale (L-1) was used to assess life satisfaction [24]. Participants answered the question “All things considered, how satisfied are you with your life these days?” on a scale ranging from 0 (not at all satisfied) to 10 (completely satisfied).

#### Health Behavior

Health behavior was assessed by asking participants questions about their health behavior within a typical week. Participants were asked about their exercise routines (On average, how many days a week do you exercise?), fruit consumption (On average, how many days a week do you eat fruit?), vegetable consumption (On average, how many days a week do you eat vegetables?), soft drink consumption (On average, how many days a week do you drink sugary soft drinks?), alcohol consumption (On average, how many days a week do you drink alcohol?), cigarette consumption (On average, how many days a week do you smoke cigarettes?), and drug consumption (On average, how many days a week do you use illegal drugs?). Participants answered the questions on scales ranging from 0 (0 days) to 7 (7 days). This study was part of a larger study, and therefore the raw data set contains further variables that have not been described because they exceed the scope of this study.

### Statistical Analyses

All statistical analyses were conducted with the statistical software SPSS (version 29.0.0.0; IBM Corp). Cronbach α was calculated for the 2 subscales of the revised German eHealth Literacy Scale to ensure the quality of the measures. Two-sided independent sample *t* tests were conducted to determine the significant differences between the subgroups. For all 2-tailed *t* tests, unequal variance was assumed. Throughout the analyses, group differences were considered significant if *P*<.05, which should be taken into consideration when interpreting the results. Interested readers can find detailed information on when it is appropriate to adjust significance thresholds (eg, disjunction testing vs conjunction testing vs individual testing) elsewhere [25]. Pearson correlations were calculated to explore the associations between digital health literacy and physical health, mental health, life satisfaction, and health behaviors. Most of the instruments used in this study relied on Likert-like scales and therefore produced ordinal data. There has been a long debate about whether data from Likert-like scales should be analyzed using parametric statistics or less sensitive and less powerful nonparametric statistics (eg, 2-sample *t* test vs Wilcoxon rank sum test, Pearson coefficient of correlation vs Spearman rank correlation) [26-28]. Previous research has demonstrated that parametric statistics can be quite robust against violations of its assumptions, especially with large sample sizes [26-28]. One article concluded that “Parametric statistics can be used with Likert data, with small sample sizes, with unequal variances, and with non-normal distributions, with no fear of ‘coming to the wrong conclusion.’” These findings are consistent with empirical literature “dating back nearly 80 years” [26]. Hence, parametric statistics were chosen for this analysis to avoid an unnecessary loss of information. The final data analyses were based on the 1996 study participants who answered all items from the revised German eHealth Literacy Scale.

### Subgroup Analysis

In Germany, the Health Literacy Survey Germany project has assessed the general health literacy of the population in Germany and relevant subgroups [8]. To simplify and facilitate comparisons between different studies, similar subgroups were chosen for this study. The subgroups were divided along the lines of level of education (low, middle, and high), social status (low, middle, and high), age (16-29 years, 30-45 years, 46-64 years, and 65 years and older), chronic disease (no and yes), migration background (no and yes), and sex (male and female). The German version of the MacArthur Scale was used to assess social status [29]. Scores were classified as low (1-4), middle (5-7), and high (8-10). Educational degrees (eg, low=no degree, middle=high school degree, and high=university degree) were used to assess participants’ level of education. Educational degrees were classified as low (ohne Haupt-/Volksschulabschluss; Haupt-/Volksschulabschluss; Mittlere Reife, Realschulabschluss, Fachschulreife; Abschluss der Polytechnischen Oberschule), middle (Fachhochschulreife, Abschluss einer Fachoberschule; Abitur, allgemeine oder fachgebundene Hochschulreife), and high (Fach-/Hochschulstudium).

## Results

### Sample Characteristics

To increase the representativeness of the sample, survey weights were calculated, and the sample was weighted accordingly. Table 1 provides invited, unweighted, and weighted sample characteristics by sex, age, state, level of education, migration background, chronic disease, and social status.

**Table 1:** Nationally representative survey of the population in Germany: invited, unweighted, and weighted sample characteristics by sex, age, state, level of education, migration background, chronic disease, and social status^a^.

Variable			Invited sample, n (%)		Unweighted sample, n (%)		Weighted sample, n (%)
**Sex**							
	Male	1988 (50.6)		955 (47.8)		978 (49)	
	Female	1939 (49.4)		1041 (52.2)		1019 (51)	
**Age (years)**							
	16-29	825 (21)		459 (23)		353 (17.7)	
	30-45	904 (23)		433 (21.7)		475 (23.8)	
	46-64	1256 (32)		598 (30)		654 (32.7)	
	65 years and older	942 (24)		506 (25.4)		514 (25.8)	
**State**							
	Schleswig-Holstein	138 (3.5)		91 (4.6)		70 (3.5)	
	Hamburg	90 (2.3)		43 (2.2)		43 (2.2)	
	Lower Saxony	381 (9.7)		208 (10.4)		191 (9.6)	
	Bremen	31 (0.8)		11 (0.6)		16 (0.8)	
	Northrhine-Westphalia	844 (21.5)		384 (19.2)		430 (21.5)	
	Hesse	298 (7.6)		166 (8.3)		151 (7.6)	
	Rhineland-Palatinate	196 (5)		88 (4.4)		99 (4.9)	
	Baden-Württemberg	534 (13.6)		300 (15)		265 (13.3)	
	Bavaria	643 (16.4)		370 (18.5)		316 (15.8)	
	Saarland	37 (0.9)		20 (1.0)		24 (1.2)	
	Berlin	176 (4.5)		89 (4.5)		88 (4.4)	
	Brandenburg	113 (2.9)		47 (2.4)		61 (3.1)	
	Mecklenburg-West Pomerania	70 (1.8)		30 (1.5)		39 (2)	
	Saxony	184 (4.7)		69 (3.5)		97 (4.9)	
	Saxony-Anhalt	98 (2.5)		43 (2.2)		53 (2.7)	
	Thuringia	94 (2.4)		37 (1.9)		52 (2.6)	
**Level of education**							
	Low	N/A^b^		1091 (54.7)		1105 (55.3)	
	Middle	N/A		399 (20)		372 (18.7)	
	High	N/A		494 (24.7)		511 (25.6)	
	Missing values	N/A		12 (0.6)		8 (0.4)	
**Migration background**							
	No	N/A		1867 (93.5)		1873 (93.8)	
	Yes	N/A		129 (6.5)		124 (6.2)	
**Chronic disease**							
	No	N/A		1257 (63)		1230 (61.6)	
	Yes	N/A		714 (35.8)		742 (37.2)	
	Missing values	N/A		25 (1.3)		25 (1.2)	
**Social status**							
	Low	N/A		312 (15.6)		315 (15.8)	
	Middle	N/A		1393 (69.8)		1385 (69.4)	
	High	N/A		291 (14.6)		296 (14.8)	

^a^Due to sample weighting and rounding, sample sizes may vary, and percentages may exceed or fall below 100%.

^b^N/A: not available.

### Quality of Measures

Using the unweighted data, Cronbach α was calculated for the 2 subscales of the revised German eHealth Literacy Scale to ensure the quality of the measures. Widely used conventions define Cronbach α values of .7 and higher as acceptable [30,31]. Both scales surpassed this widely used Cronbach α threshold (information seeking: .897 and information appraisal: .839).

### Level of Education

On average, people with a high level of education had higher perceived competency levels in the domains of information seeking (*t*_3.415_=1068.213; *P*<.001; *d*=0.177) and information appraisal (*t*_6.406_=1118.823; *P*<.001; *d*=0.327) than people with a low level of education. Perceived competency levels did not differ significantly between people with a high level of education and people with a middle level of education (information seeking: *t*_1.062_=809.769; *P*=.29; *d*=0.072 and information appraisal: *t*_0.434_=818.391; *P*=.66; *d*=0.029). Furthermore, perceived competency levels in the domain of information seeking did not differ significantly between people with a middle level of education and people with a low level of education (*t*_1.926_=701.099; *P*=.05; *d*=0.110). However, people with a middle level of education had higher perceived competency levels in the domain of information appraisal than people with a low level of education (*t*_5,456_=750.726; *P*<.001; *d*=0.301).

### Social Status

On average, people with a high social status had higher perceived competency levels in the domains of information seeking (*t*_4.947_=606.910; *P*<.001; *d*=0.399) and information appraisal (*t*_6.583_=598.474; *P*<.001; *d*=0.530) than people with a low social status. Furthermore, people with a high social status had higher perceived competency levels than people with a middle social status (information seeking: *t*_2.222_=437.791; *P*=.03; *d*=0.140 and information appraisal: *t*_2.751_=460.093; *P*=.006; *d*=0.166). Additionally, people with a middle social status had higher perceived competency levels than people with a low social status (information seeking: *t*_4.089_=438.922; *P*<.001; *d*=0.271 and information appraisal: *t*_5.526_=437.177; *P*<.001; *d*=0.368).

### Age

On average, perceived competency levels in the domain of information seeking did not differ significantly between people aged 16-29 years and people aged 65 years and older (*t*_1.889_=826.226; *P*=.06; *d*=0.127). However, people aged 16-29 years had higher perceived competency levels in the domain of information appraisal than people aged 65 years and older (*t*_4.787_=831.799, *P*<.001, *d*=0.320). People aged 16-29 years had higher perceived competency levels than people aged 46-64 years (information seeking: *t*_2.253_=761.604; *P*=.02; *d*=0.146 and information appraisal: *t*_4.002_=783.957; *P*<.001; *d*=0.257). Perceived competency levels did not differ significantly between people aged 16-29 years and people aged 30-45 years (information seeking: *t*_–0.256_=754.114; *P*=.80; *d*=–0.018 and information appraisal: *t*_–0.184_=762.075; *P*=.85; *d*=–0.013). People aged 30-45 years had higher perceived competency levels than people aged 46-64 years (information seeking: *t*_2.764_=1062.057; *P*=.006; *d*=0.165 and information appraisal: *t*_4.542_=1069.427; *P*<.001; *d*=0.270) and people aged 65 years and older (information seeking: *t*_2.299_=979.125; *P*=.02; *d*=0.145 and information appraisal: *t*_5.301_=979.619; *P*<.001; *d*=0.335). However, perceived competency levels did not differ significantly between people aged 46-64 years and people aged 65 years and older (information seeking: *t*_–0.182_=1048.014; *P*=.86; *d*=–0.011 and information appraisal: *t*_1.236_=1058.857; *P*=.22; *d*=0.074).

### Chronic Disease

On average, perceived competency levels in the domains of information seeking (*t*_–0.367_=1548.068; *P*=.71; *d*=–0.017) and information appraisal (*t*_1.538_=1512.596; *P*=.12; *d*=0.072) did not differ significantly between people without a chronic disease and people with a chronic disease.

### Migration Background

On average, perceived competency levels in the domains of information seeking (*t*_0.368_=139.851; *P*=.71; *d*=0.034) and information appraisal (*t*_–0.604_=142.612; *P*=.55; *d*=–0.052) did not differ significantly between people without a migration background and people with a migration background.

### Sex

On average, women had higher perceived competency levels in the domain of information seeking than men (*t*_2.333_=1992.953; *P*=.02; *d*=0.104). However, perceived competency levels in the domain of information appraisal did not differ significantly between women and men (*t*_0.016_=1993.874; *P*=.99; *d*=0.001). Table 2 shows the digital health literacy of the population in Germany by level of education, social status, age, chronic disease, migration background, and sex.

**Table 2:** Nationally representative survey of the population in Germany: digital health literacy by the level of education, social status, age, chronic disease, migration background, and sex. Statistically significant differences are indicated by superscript letters.

Variable		Information seeking, mean (SD)	Information appraisal, mean (SD)
**Population in Germany**			
	Overall	3.7940 (0.92522)	3.7680 (0.85482)
**Level of education**			
	Low (a)	3.7323^(c)^ (0.95846)	3.6483^(b), (c)^ (0.89606)
	Middle (b)	3.8350 (0.86613)	3.9076^(a)^ (0.75513)
	High (c)	3.8983^(a)^ (0.88436)	3.9303^(a)^ (0.78663)
**Social status**			
	Low (d)	3.5640^(e), (f)^ (0.99864)	3.4817^(e), (f)^ (0.93104)
	Middle (e)	3.8148^(d), (f)^ (0.90731)	3.7974^(d), (f)^ (0.84026)
	High (f)	3.9414^(d), (e)^ (0.88624)	3.9347^(d), (e)^ (0.76645)
**Age (years)**			
	16-29 (g)	3.8649^(i)^ (0.86732)	3.9073^(i), (j)^ (0.77874)
	30-45 (h)	3.8804^(i), (j)^ (0.85653)	3.9174^(i), (j)^ (0.78404)
	46-64 (i)	3.7329^(g), (h)^ (0.92275)	3.6939^(g), (h)^ (0.85758)
	65 years and older (j)	3.7433^(h)^ (1.01659)	3.6286^(g), (h)^ (0.92788)
**Chronic disease**			
	No (k)	3.7915 (0.92113)	3.7943 (0.84147)
	Yes (l)	3.8073 (0.93183)	3.7326 (0.87641)
**Migration background**			
	No (m)	3.7960 (0.92588)	3.7652 (0.85894)
	Yes (n)	3.7646 (0.91835)	3.8099 (0.79211)
**Sex**			
	Male (o)	3.7448^(p)^ (0.91756)	3.7677 (0.84414)
	Female (p)	3.8413^(o)^ (0.93051)	3.7683 (0.86538)

### Correlates of Information Seeking

Participants’ perceived competency levels in the domain of information seeking were weakly positively and significantly correlated with their physical health, mental health, life satisfaction, exercise routines, fruit consumption, and vegetable consumption. Furthermore, information seeking was weakly negatively and significantly correlated with alcohol consumption. There were no significant correlations between information seeking and soft drink consumption, cigarette consumption, and drug consumption. Table 3 provides further information about the correlation coefficients and their significance levels.

**Table 3:** Nationally representative survey of the population in Germany: correlations (Pearson r and 2-tailed *P* value) between digital health literacy (information seeking and information appraisal) and physical health, mental health, life satisfaction, and health behaviors.

		Information seeking	Information appraisal	Physical health	Mental health	Life satisfaction	Exercise routine	Fruit consumption	Vegetable consumption	Soft drink consumption	Alcohol consumption	Cigarette consumption	Drug consumption
**Information seeking**													
	*r*	1											
	*P* value	—^a^											
**Information appraisal**													
	*r*	0.794^b^	1										
	*P* value	<.001	—										
**Physical health**													
	*r*	.099^b^	0.150^b^	1									
	*P* value	<.001	<.001	—									
**Mental health**													
	*r*	0.109^b^	0.156^b^	0.510^b^	1								
	*P* value	<.001	<.001	<.001	—								
**Life satisfaction**													
	*r*	0.126^b^	0.157^b^	0.528^b^	0.698^b^	1							
	*P* value	<.001	<.001	<.001	<.001	—							
**Exercise routines**													
	*r*	0.070^b^	0.096^b^	0.216^b^	0.123^b^	0.132^b^	1						
	*P* value	.002	<.001	<.001	<.001	<.001	—						
**Fruit consumption**													
	*r*	0.061^b^	0.050^c^	0.077^b^	0.124^b^	0.127^b^	0.224^b^	1					
	*P* value	.007	.026	<.001	<.001	<.001	<.001	—					
**Vegetable consumption**													
	*r*	0.099^b^	0.145^b^	0.084^b^	0.085^b^	0.111^b^	0.218^b^	0.485^b^	1				
	*P* value	<.001	<.001	<.001	<.001	<.001	<.001	<.001	—				
**Soft drink consumption**													
	*r*	–0.036	–0.026	–0.056*	–0.088^b^	–0.100^b^	–0.120^b^	–0.190^b^	–0.161^b^	1			
	*P* value	.104	.251	.012	<.001	<.001	<.001	<.001	<.001	—			
**Alcohol consumption**													
	*r*	–0.049*	–0.019	0.073^b^	0.126^b^	0.120^b^	–0.018	–0.023	0.024	–0.085^b^	1		
	*P* value	.028	.389	.001	<.001	<.001	.414	.305	.285	<.001	—		
**Cigarette consumption**													
	*r*	–0.011	–0.037	–0.112^b^	–0.101^b^	–0.132^b^	–0.136^b^	–0.129^b^	–0.061^b^	0.100^b^	.000	1	
	*P* value	.626	.102	<.001	<.001	<.001	<.001	<.001	.007	<.001	1.000	—	
**Drug consumption**													
	*r*	–0.015	.000	0.009	–0.031	–0.042	0.054^c^	–0.010	0.017	0.090^b^	–0.004	0.152^b^	1
	*P* value	.514	.984	.682	.166	.059	.016	.659	.457	<.001	.860	<.001	—

^a^Not applicable.

^b^Correlation is significant at the .01 level (2-tailed).

^c^Correlation is significant at the .05 level (2-tailed).

### Correlates of Information Appraisal

Participants’ perceived competency levels in the domain of information appraisal were weakly positively and significantly correlated with their physical health, mental health, life satisfaction, exercise routines, fruit consumption, and vegetable consumption. There were no significant correlations between information appraisal and soft drink consumption, alcohol consumption, cigarette consumption, and drug consumption. Table 3 provides further information about the correlation coefficients and their significance levels.

## Discussion

### Principal Findings

To guide the development of target group–specific digital health literacy interventions, it is necessary to know the digital health literacy of different subgroups within a society. For Germany, such data have not been collected with appropriate and methodological sound measures. Therefore, this study representatively assessed the digital health literacy of the population in Germany and relevant subgroups. The results suggest that people with a low level of education and people with a low social status would benefit from digital health literacy interventions that address their competencies in the domains of information seeking and information appraisal. These findings align with previous research, which suggests that individuals with lower levels of education and lower social status tend to have lower general health literacy [8,32]. Furthermore, the results suggest that older people would likely benefit from digital health literacy interventions that address their competencies in the domains of information seeking and information appraisal. Once again, these findings are in line with results from general health literacy research, which highlights that older individuals may constitute a vulnerable group deserving special attention [8,32,33].

Previous research has found that people with chronic diseases and people with migration backgrounds have lower health literacy levels [8,34,35]. Such differences, however, were not found in this study. People with a chronic disease and people without a chronic disease did not significantly differ in regard to their digital health literacy. Neither did people with a migration background and people without a migration background significantly differ in regard to their digital health literacy. The last finding is in line with results from a study conducted in Israel, which suggests that the migration status of an individual may be linked to general health literacy but not necessarily to digital health literacy [36]. The authors of the study hypothesize that this finding might be attributed to the fact that certain language barriers are less prominent on the internet compared to real-world settings. Another contributing factor could be that the study sample might have contained individuals with migration backgrounds who possess particularly strong language skills. Regarding sex, this study suggests that men might benefit from digital health literacy interventions that specifically address their competencies in the domain of information seeking. These findings corroborate earlier research results which indicate that women have higher (digital) health literacy than men, even though the differences are often small [7,8,37].

Besides comparing the digital health literacy of different societal groups, this study explored the associations between digital health literacy and diverse health-related constructs and behaviors. The results suggest that digital health literacy is weakly positively correlated with physical health, mental health, life satisfaction, exercise routines, fruit consumption, and vegetable consumption. Moreover, higher perceived competency levels in the domain of information seeking were weakly negatively correlated with alcohol consumption. When interpreting these results, however, it must be stressed that the found associations were rather weak.

In line with previous research, information appraisal was more strongly associated with mental health, physical health, and life satisfaction than information seeking [9]. Interestingly, the results suggest that digital health literacy is more strongly associated with health perceptions (eg, mental health) than with health behaviors (eg, exercise routines). As a reviewer (EN) of this paper pointed out, this finding might be explained by methodological considerations. For example, 2 health perception variables (eg, “Overall, how do you currently rate your mental health?”) might show stronger associations with each other than a health perception variable and a health behavior variable (eg, “On average, how many days a week do you exercise?”). Future studies should develop adequate research designs to test this hypothesis.

### Limitations and Future Directions

The results of this study demonstrate the importance of digital health literacy in society. There are, however, limitations that must be kept in mind when interpreting the results. Three of these limitations seem especially important and need to be discussed in more detail. The first limitation concerns the study design. This study used a cross-sectional study design, and therefore no causal inference can be drawn [23]. This study found, for example, that participants’ digital health literacy is positively and significantly correlated with their physical health. From this finding, however, one cannot conclude that digital health literacy causes physical health. To draw a causal inference like this, future studies could, for example, use an experimental study design and actively manipulate the digital health literacy of the study participants before assessing their physical health [38].

The second limitation concerns the data collection method of this study. Data collection took place via a web-based survey platform. Hence, participants needed basic technical skills and access to the internet to participate in the study. It is known, however, that older people use the internet less frequently than younger people [39]. Therefore, this study can only be representative of the German population with internet access. People without any experience with the internet probably have lower digital health literacy scores. Hence, the data collection method should be taken into account when comparing the results of this study to other digital health literacy studies that might have used other data collection methods such as in-person or telephone interviews. There is another problem that might have affected the representativeness of the study results, which has already been raised in the survey methodology section and concerns the nonresponders and potential selection bias. As a reminder, 50.6% (1988/3927) of the invited sample were men, but only 47.8% (955/1996) of the unweighted sample were men. After the weighting procedure, 49% (978/1997) of the sample were counted as men. During the analyses, it was found that, on average, women had higher perceived competency levels in the domain of information seeking than men. One might argue that men with an above-average interest in the topic of digital health literacy were more likely to complete the study, and their response data were weighted. Hence, the found difference between women and men might be even more pronounced within the German population. Such an argument can also be made regarding other demographic factors. Since we have no information about the digital health literacy of the nonresponders, we currently cannot test this hypothesis. Nevertheless, it is important to bear in mind that there were demographic differences between responders and nonresponders, as can be seen in Table 1, and this might have affected the results, even after the weighting procedure.

The third limitation concerns the types of measures that were used in this study. Besides multi-item measures, this study also used single-item measures. Single-item measures are often criticized because they usually cannot assess complex constructs (eg, personality) and may be less reliable under specific circumstances [40-42]. In many situations, however, there are good arguments in favor of the use of single-item measures, and previous research has shown that these measures can be reliable and valid [40,43,44]. Nevertheless, it would be interesting to substitute the single-item measures with multi-item measures in future studies to investigate whether these changes would alter the general direction of the results. Furthermore, this study also relied on self-report measures. Self-report measures, however, are often criticized because they may produce inaccurate results [45-47]. When asked about their vegetable consumption within a typical week, for example, respondents might give an inaccurate answer because they usually do not track their eating habits very carefully. When asked about their drug consumption within a typical week, respondents might be inclined to lie because they do not want to make a bad impression or admit to themselves that they might have a drug problem. Therefore, to verify these results, it would be interesting to repeat this study with behavioral, observational, and performance measures instead of self-report measures.

### Conclusions

Overall, the results of this study demonstrate that digital health literacy is associated with diverse health outcomes and behaviors. Furthermore, the results provide a starting point for the development of target group–specific digital health literacy interventions.

## Abbreviations

Forsa: forsa Gesellschaft für Sozialforschung und statistische Analysen mbH

## Data Availability

The data sets generated and analyzed during this study are available from the independent, nonprofit foundation Stiftung Gesundheitswissen on reasonable request.
